# Presentation of massive orbital hidrocystoma at birth: case report and review of the literature

**DOI:** 10.1186/s40662-017-0069-7

**Published:** 2017-02-23

**Authors:** Bahram Eshraghi, Mohammad-Ali Abtahi, Seyed Ali Sonbolastan, Zahra Kasaie, Seyed-Hossein Abtahi

**Affiliations:** 1 Ophthalmology Department, School of Medicine, Tehran University of Medical Sciences, Tehran, Iran; 2 Ophthalmology Department, School of Medicine, Isfahan University of Medical Sciences, Isfahan, Iran; 3 Isfahan Eye Research Center, Isfahan University of Medical Sciences, Feiz Hospital, Qods Sq., Isfahan, Iran; 4 Pathology Department, School of Medicine, Isfahan University of Medical Sciences, Isfahan, Iran

**Keywords:** Orbital hidrocystoma, Pediatric orbital tumor, Sudoriferous cyst

## Abstract

**Background:**

Hidrocystoma, or sudoriferous cyst, is an eyelid tumor originating from apocrine or eccrine sweat glands. Its presence in the orbit is relatively rare.

**Case presentation:**

A full-term female child with severe right eye extrusion was referred to our department two hours after birth. We performed cyst aspiration under ultrasonic guidance; 15 cc straw-colored fluid was obtained and proptosis resolved significantly. Orbital magnetic resonance imaging (MRI) showed a large unilocular retrobulbar mass with hypo-intensity in T1 and hyper-intensity in T2. The case underwent further daily ocular assessment until day 5; when proptosis began to worsen again. On day 6, under general anesthesia, we performed aspiration and then the cyst was completely removed with an intact wall through a trans-conjunctival incision. The diameter of the aspirated cyst was about 4 cm. In pathologic consultation, a cystic cavity lined by a layer of sweat duct like epithelium with apical snouts consistent with the diagnosis of apocrine hidrocystoma was reported.

**Conclusion:**

To date, in the literature, six other cases of orbital hidrocystoma have been reported in childhood with protean clinical pictures; none of which presented at birth. Herein, we introduce the first case report at birth and also provide a review on the literature. Our report strongly argues against the well reputed theory of traumatic origin for orbital hidrocystoma; it has been postulated that this tumor may be the result of sweat gland cells implantation through the orbit. We thereby suggest the possible presence of choristomatous ectopic sweat gland cells in the orbit during embryogenesis.

## Background

Hidrocystoma, or sudoriferous cyst, is an eyelid tumor originating from apocrine or eccrine sweat glands [1]. Presentation of this tumor in the orbit is very rare [2–6]. In 1973, Saunders reported a superficial 2 mm mass in the superior orbit of a newborn [2]. To date, six other cases have been reported in childhood with protean clinical findings; none of these presented at birth [2–6]. In this report, we describe a massive hidrocystoma at birth and review the related literature.

## Case presentation

A full-term, female child with severe right eye proptosis was referred to our department in central Iran two hours after birth by Caesarian section. The baby had a birth weight of 3410 g and Apgar scores of 10 at one and five minutes. She was the second child of a 30-year old healthy mother who had undergone cesarean section for her first child as well. History of trauma and amniocentesis or similar procedures during the pregnancy period was negative. Neither the parents nor the older brother had a history of systemic or ocular problems.

**Fig. 1:**
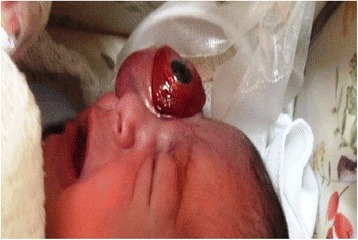
Severe right eye extrusion on admission

**Fig. 2:**
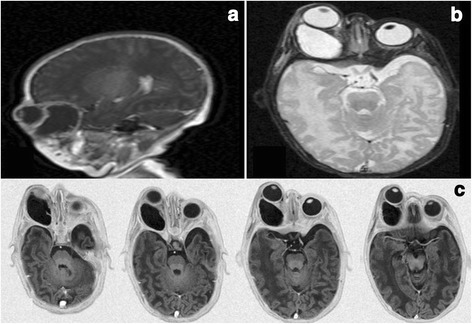
A large unicystic retrobulbar mass with hypo-intensity in T1 and hyper-intensity in T2 (**a**: Sagittal T1view, **b**: Axial T2 view, **c**: axial T1 views)

**Fig. 3:**
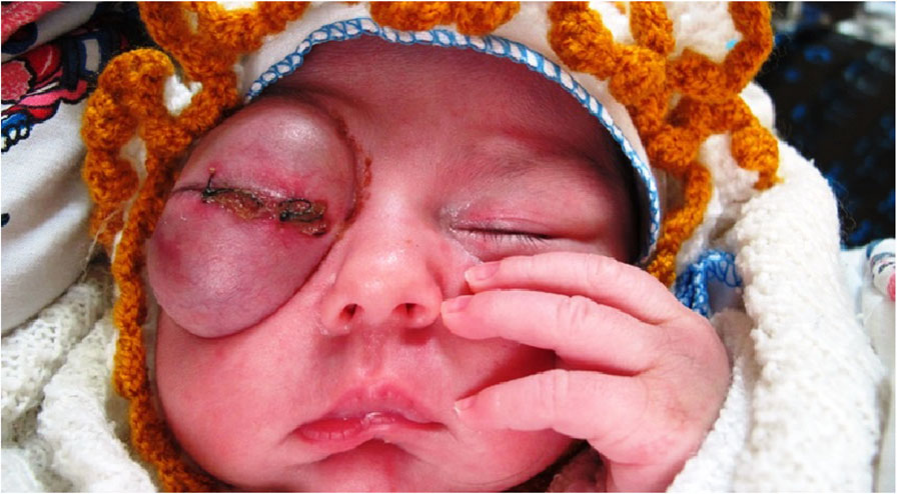
The patient’s presentation on the 5th day; proptosis began to worsen again

**Fig. 4:**
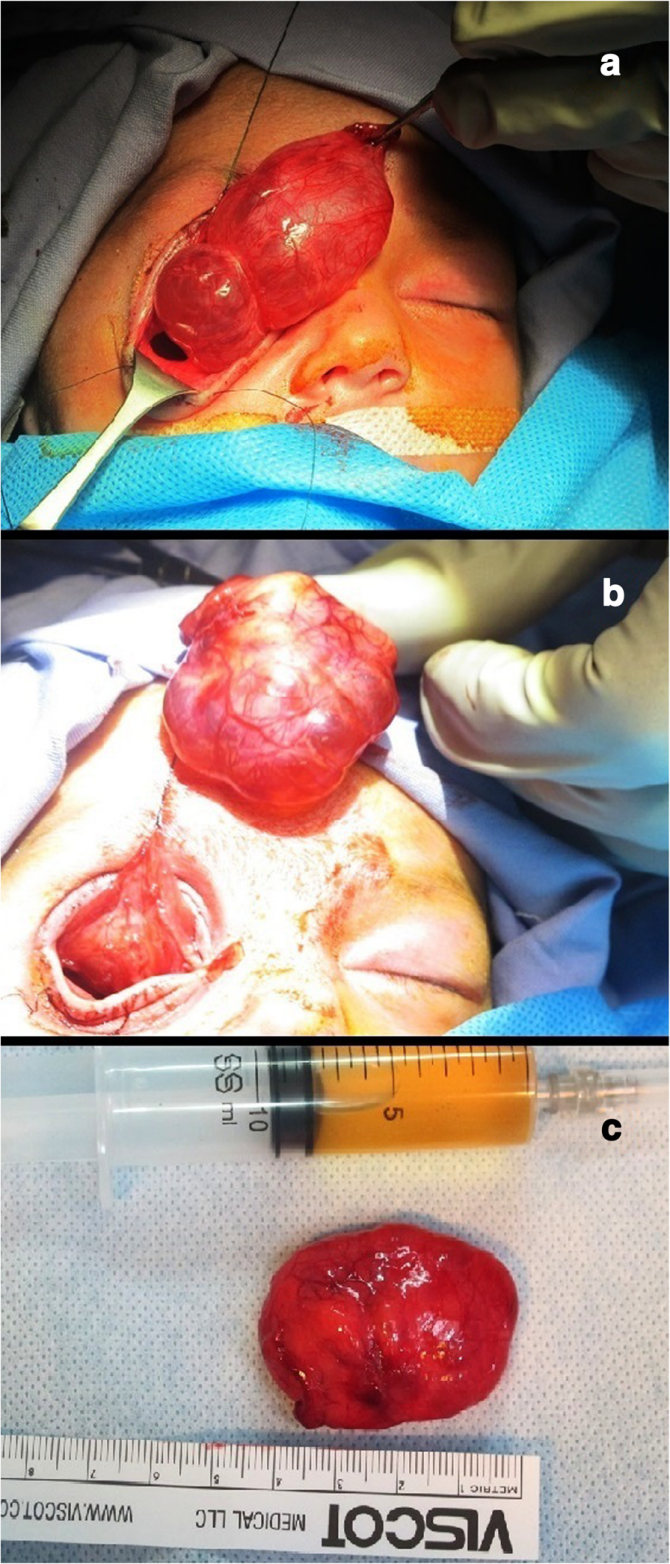
Excisional biopsy of the cyst on the 6th day. **a**, **b**: During the surgery, **c**: The excised cyst and the aspirated straw colored fluid)

**Fig. 5:**
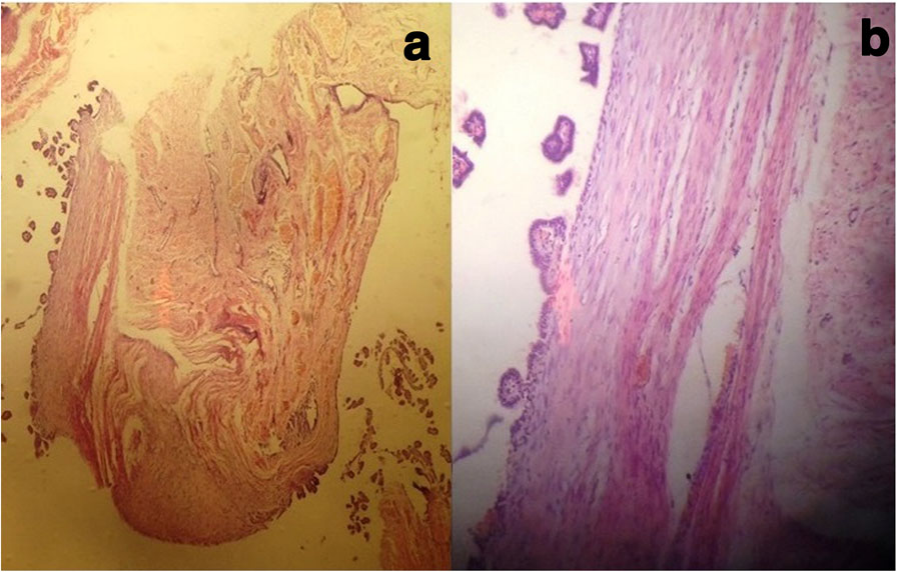
Histopathologic examination; **a** and **b**: a cystic cavity lined by a layer of sweat duct like epithelium (flat to cuboid) with typical apical snouts consistent with the diagnosis of apocrine hidrocystoma was reported (Hematoxylin and eosin stain)

**Fig. 6:**
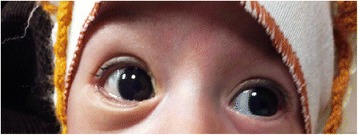
After 3 months of follow-up

Under ocular examination, she had severe right eye extrusion (Fig. 1) and a total corneal epithelial defect with a good red reflex. In palpation and transillumination, the right orbit seemed to include a cystic mass with fluid. Both pupils were reactive to light and no relative afferent pupillary defect was detectable. Anterior/posterior segment examination of the left eye was unremarkable. Based on the opinion of the consulting neonatologist regarding the general conditions of the case, general anesthesia and imaging was postponed to at least 72 h later. We admitted the case in our ward and performed cyst aspiration under ultrasonic guidance; 15 cc straw-colored fluid was obtained and proptosis resolved significantly. Then, blepharorrhaphy was performed due to eyelids eversion and severe conjunctival chemosis. Appropriate topical drugs were administered and the patient referred again to the neonatology department for further stabilization and evaluation. Orbital magnetic resonance imaging (MRI) showed a large retro-orbital mass being unilocular and extraconal with hypo-intensity in T1 and hyper-intensity in T2 (Fig. 2). The case underwent further daily ocular assessment until day 5; when proptosis began to worsen again (Fig. 3). On day 6, under general anesthesia, we aspirated the mass again and then, through a trans-conjunctival incision 3 mm beneath the tarsal plate, the mass was exposed. It was located in the inferior aspect of the orbit extending to its apex. Hemorrhagic vessels were cauterized and by blunt and sharp dissection, the mass was completely removed en-bloc. The wound was closed with vicryl 8.0. The diameter of the aspirated mass was about 4 cm (Fig. 4). In pathologic consultation, a cystic cavity lined by a layer of sweat duct like epithelium with apical snouts consistent with the diagnosis of apocrine hidrocystoma was reported (Fig. 5). The patient was followed for 3 months (Fig. 6) and had no significant complications.

## Discussion

Presentation of hidrocystoma in the orbit is extremely rare to the extent that its presentation at birth, as a micro-cyst had only been reported once [2]. The point that makes our case very impressive was its massive size, orbital location and presentation at birth.

As mentioned earlier, there are 7 other known cases of orbital hidrocystoma in childhood [2–6] (Table 1). Of the 8 existing cases, 5 presented before the age of 1 while 3 cases presented at later ages. Tumor types were apocrine in 6 and eccrine in 2 cases. We did not have access to the full-text version of one of these cases [3]. Of 7 other known orbital locations, 3 were superficial and 4 were deep [2, 4–6]. Only one case had a history of significant orbital trauma [6]. Origin sites were medial (3 cases), superior (1 case), supra-temporal (1 case), inferior (1 case) and retro-bulbar (1 case). Computed tomography (CT) scan may demonstrate bone remodeling with no erosion. The MRI signal was reported as hypo- or iso-intense in T1 and hyper-intense in T2 [2–6].

**Table 1:** Reported cases of orbital hidrocystoma in childhood

Case number	Author(s)	Year	Patient Age; Sex	Presentation symptoms	Cyst size	Subtype	Radiologic features
1	Saunders [2]	1973	At birth; Male	Medial superficial orbital mass, without globe displacement no visual axis involvement	2 mm	Apocrine	CT: cyst without bone erosion
2	Mims et al. [3]	1977	NA	NA	NA	Apocrine	NA
3	Haider et al. [4]	2005	4 months; Male	Inferior deep orbital mass with superior globe displacement	Large	Apocrine	CT: slight bone remodeling MRI: hypointense in T1 and hyperintense in T2
4	Chung et al. [5]	2007	20 days; Male	Medial deep orbital mass with lateral globe displacement	About 1.3 cm	Apocrine	MRI: hypointense in T1 and hyperintense in T2
5	Malihi et al. [6]	2015	8 y/o; Male	Supra-temporal deep orbital mass with inferior globe displacement and 2 mm proptosis, no diplopia or visual loss	Large	Eccrine	CT: erosion of lateral orbital wall MRI: Isointense in T1 and hyperintense in T2
6	Malihi et al. [6]	2015	13 y/o; Female	Superior superficial orbital mass without globe displacement, no diplopia, proptosis or visual loss, history of significant blunt trauma	Large	Apocrine	MRI: Isointense in T1 and hyperintense in T2
7	Malihi et al. [6]	2015	2 months; Male	Medial canthal superficial mass, no other symptoms	Small	Eccrine	Not performed
8	Present case	2016	At birth; Female	Deep retro-orbital mass, globe extrusion	Huge	Apocrine	MRI: hypointense in T1 and hyperintense in T2

*NA* = not available; *y/o* = years old; *CT* = Computed Tomography; *MRI* = Magnetic Resonance Imaging

From a pathological standpoint, hidrocystoma is a benign cyst originating from a sweat gland apocrine or eccrine in nature. The apical part of cellular cytoplasm is decapitated in the apocrine type (decapitation), while it remains intact in eccrine (exocytosis) glands [6]. This difference in secretion mechanisms gives the pathologic appearance of apical ‘snouts’ in the apocrine type.

In a review by Shield and Shield, the authors believe that differentiating between the main causes of orbital cysts without bone involvement in childhood may not be possible even with CT scan or MRI [7]. Differential diagnosis of what we presented in our neonate case may include: (i) surface epithelial lesions (e.g., conjunctival, respiratory epithelial, epidermal and dermoid cysts); (ii) teratomas; (iii) Neural cysts (e.g., cephalocele, congenital cystic eye); (iv) originating from adjacent structures (e.g., mucocele and dentigerous cysts); (v) parasitic cysts [7].

## Conclusions

The picture of our case strongly argues against the well reputed theory of traumatic origin for orbital hidrocystoma by which it was postulated that this tumor may be the result of sweat gland cells implantation through the orbit [6]. Presentation of a large deep orbital hidrocystoma at birth in our case suggests the possible presence of choristomatous ectopic sweat gland cells in the orbit during embryogenesis.

Taken together, orbital hidrocystoma should be considered as a differential diagnosis of small/large; superficial/deep; congenial/post-traumatic; and, childhood/adult orbital cysts.
